# CD4^+ ^CD25^+ ^FoxP3^+ ^regulatory T cells suppress cytotoxicity of CD8^+ ^effector T cells: implications for their capacity to limit inflammatory central nervous system damage at the parenchymal level

**DOI:** 10.1186/1742-2094-9-41

**Published:** 2012-02-28

**Authors:** Kerstin Göbel, Stefan Bittner, Nico Melzer, Susann Pankratz, Angela Dreykluft, Michael K Schuhmann, Sven G Meuth, Heinz Wiendl

**Affiliations:** 1 Department of Neurology - Inflammatory Disorders of the Nervous System and Neurooncology, University of Muenster, Albert-Schweitzer-Campus 1, 48149 Muenster, Germany; 2 Institute of Physiology - Neuropathophysiology, University of Muenster, Robert-Koch-Strasse 27a, 48149 Muenster, Germany

**Keywords:** CD4^+ ^T regulatory cells, CD8 T effector cells, CNS parenchyma, Cytotoxicity, Neuroinflammation

## Abstract

**Background:**

CD4^+ ^CD25^+ ^forkhead box P3 (FoxP3)^+ ^regulatory T cells (T reg cells) are known to suppress adaptive immune responses, key control tolerance and autoimmunity.

**Methods:**

We challenged the role of CD4^+ ^T reg cells in suppressing established CD8^+ ^T effector cell responses by using the OT-I/II system *in vitro *and an OT-I-mediated, oligodendrocyte directed *ex vivo *model (ODC-OVA model).

**Results:**

CD4^+ ^T reg cells dampened cytotoxicity of an ongoing CD8^+ ^T effector cell attack *in vitro *and within intact central nervous system tissue *ex vivo*. However, their suppressive effect was limited by the strength of the antigen signal delivered to the CD8^+ ^T effector cells and the ratio of regulatory to effector T cells. CD8^+ ^T effector cell suppression required T cell receptor-mediated activation together with costimulation of CD4^+ ^T reg cells, but following activation, suppression did not require restimulation and was antigen non-specific.

**Conclusions:**

Our results suggest that CD4^+ ^T reg cells are capable of suppressing CD8^+ ^T effector cell responses at the parenchymal site, that is, limiting parenchymal damage in autoimmune central nervous system inflammation.

## Background

Naturally occurring CD4^+ ^CD25^+ ^regulatory T cells (T reg cells) expressing the transcription factor forkhead box P3 (FoxP3) are continuously produced in the thymus and are essential for the maintenance of peripheral immunological self-tolerance and the control of a variety of physiological and pathological immune responses [1,2].

Depletion of T reg cells or mutations in the FoxP3 gene lead to spontaneous autoimmune disease *in vivo *[3,4]. *In vitro *coculture experiments demonstrate that naturally occurring T reg cells potently suppress proliferation and cytokine secretion of naïve CD4^+ ^and CD8^+ ^T cells upon stimulation with a specific antigen or with a polyclonal T cell receptor (TCR) stimulator in the presence of antigen-presenting cells (APCs) for costimulation in a cell-cell contact-dependent manner [5,6]. Moreover, induction of the FoxP3 gene, which is considered to control the expression of key molecules mediating suppression, is capable of converting naïve CD4^+ ^CD25^- ^T cells into (inducible) CD4^+ ^CD25^+ ^T reg cells with suppressive function *in vivo *and *in vitro *[7,8].

T reg cells can operate at different levels during the initiation and execution of an immune response. The suppressive effects of T reg cells on the initiation of an adaptive (auto)immune response in the peripheral lymphoid compartment are well known. However, their possible impact on an ongoing T cell response at the effector site is much less clear [4]. Considering modulation of T reg cells as a potential strategy for therapeutic intervention in established autoimmune central nervous system (CNS) disorders, knowledge on the potential of T reg cells in suppressing T effector cell responses would be mandatory [1].

In the present work we challenge the role of T reg cells in suppressing established CD8^+ ^T effector cell responses, by using the OT-I/II system of ovalbumin peptide (OVA) reactive CD8^+ ^and CD4^+ ^T cells [9,10] in coculture experiments *in vitro *and in brain slice cultures from transgenic mice selectively expressing ovalbumin as a cytosolic neo-self antigen in oligodendrocytes under the control of a truncated myelin basic protein (MBP) promoter (ODC-OVA mice, [11-14]) *ex vivo*. Our results suggest that CD4^+ ^T reg cells can modulate antigen-specific CD8^+ ^T effector cell functions at the parenchymal level within intact CNS tissue in an antigen non-specific fashion.

## Materials and methods

### Mice

Wild-type C57BL/6, ODC-OVA [11], OT-I [10], as well as OT-II [9] mice were kept under pathogen-free conditions and had access to food and water ad libitum. All experiments were conducted according to the German law of animal protection and were approved by local authorities.

### T cell isolation, culture and stimulation

Isolation and stimulation of OT-I, wild-type and OT-II T reg cells was performed as previously described. Briefly, spleens were removed and single cell suspensions were generated by mashing spleens through a 40 μm strainer followed by lysis of red blood cells with ACK buffer. Splenocytes were cultured in Dulbecco's modified Eagle medium (DMEM; BioWhittaker, Verviers, Belgium) supplemented with 5% fetal calf serum (FCS; PAA Laboratories, Pasching, Germany), 10 mM 4-(2-hydroxyethyl)-1-piperazineethanesulfonic acid (HEPES; Gibco, Invitrogen, Darmstadt, Germany), 2 mM L-glutamine (PAA Laboratories), 50 μM 2-mercaptoethanol (Gibco, Invitrogen), 1% non-essential amino acids (BioWhittaker) and 25 μg/ml gentamicin (Gibco, Invitrogen).

OT-I splenocytes were plated at a density of 1 × 10^7 ^cells/well on a 12-well plate and primed by incubation for 5 days with OVA_257-264 _(SIINFEKL; Genescript, Hamburg, Germany; 1 nM) and interleukin (IL)-2 (200 IU/ml, Pepro Tech, Hamburg, Germany). After 4 days, IL-2 at a concentration of 200 IU/ml was added again to the medium. Subsequent to stimulation, H-2 K^b^-restricted OT-I T cells were purified from the splenocyte suspension using the mouse CD8^+ ^T cell isolation kit (Miltenyi, Bergisch Gladbach, Germany) following the manufacturer's instructions and yielding a purity of > 95%.

H-2 IA^b^-restricted CD4^+ ^CD25^+ ^T cells were purified from the OT-II and wild-type splenocyte suspensions using the mouse CD4 CD25 T cell isolation kit (Miltenyi) following the manufacturer's instructions. Overnight stimulation of OT-II and wild-type T reg cells was performed with 1 μg/ml anti-CD3 (immobilized) and 1 μg/ml anti-CD28 (soluble).

T cell activation status was regularly assessed by flow cytometry using the following antibodies (all by BD Bioscience, Heidelberg, Germany): rat anti-mouse CD4-PerCP (no. 553052), rat anti-mouse CD8a-PE (no. 553033), rat anti-mouse CD25-FITC (no. 554071), rat anti-mouse Alexa Fluor 647-FoxP3 (no. 560401). Flow cytometry was performed using a FACS-Calibur system (BD Bioscience) and CellQuest Pro Software (BD Bioscience).

### Cocultivation experiments

In one set of experiments, OT-I splenocytes were stimulated with OVA_257-264 _for 5 days as described above with or without wild-type or OT-II T reg cells at different concentrations. Proliferating OT-I T cells were identified by prelabeling with Cell Proliferation Dye eFluor 670 (eBioscience, Frankfurt, Germany). OT-I T cell maturation markers were assessed by flow cytometry using rat anti-mouse CD8a-PerCP (no. 553036), rat anti-mouse CD44-FITC (no. 553133), rat anti-mouse CD62L-APC (no. 553152) and rat anti-mouse CD11a-PE (no. 553121) and appropriate isotype controls (all by BD Bioscience). The relation of OT-I T cells to wild-type or OT-II T reg cells was assessed by flow cytometry after cocultivation using rat anti-mouse CD4-PerCP (no. 553052) and rat anti-mouse CD8a-PE (no. 553033).

### Cytotoxicity experiments

PKH26 (Sigma-Aldrich, Seelze, Germany) labeled EG.7 [15] and EL-4 cells [16] were used as target cells for stimulated OT-I T cells. Cleaved caspase-3^+ ^cells were identified after an incubation period of 6 h as early apoptotic cells measured by flow cytometry using Alexa Fluor 488-labeled rabbit anti-mouse cleaved caspase-3 (Cell Signaling, Danvers, MA, USA). OT-II T reg cells or wild-type T reg cells and OVA_257-264 _(SIINFEKL) were added at different concentrations as indicated.

Alternatively, 50 000 EL-4 cells/well were grown on white 96-well microassay plates (Greiner Bio-One, Frickenhausen, Germany) and incubated with different concentrations of OVA_257-264 _for 24 h. Afterwards, EL-4 cells were cocultured with 50.000 activated OT-I T cells (1:1) or 25.000 OT-II T reg cells (2:1) per well either alone or in combination for additional 6 h. The amount of ATP in the supernatant following cell lysis was assessed as a parameter of cell viability using the ATPLite™ Luminescence Assay System (PerkinElmer, Rodgau-Jügesheim, Germany) according to the manufacturer's instructions. Luminescence was measured on a Topcount NXT (PerkinElmer). Experiments were performed in triplicate.

### Preparation of acute brain slices and coculture experiments with CD8^+ ^T cells

Preparation of acute brain slices was performed following established procedures as described before [13] using naïve 6 to 10-week-old transgenic ODC-OVA mice [11]. Brain slices were incubated alone, with 5 × 10^5 ^activated OT-I T cells per slice alone or in combination with 2.5 × 10^5 ^wild-type T reg cells or 2.5 × 10^5 ^OT-II T reg cells per slice, respectively. For upregulation of major histocompatibility complex (MHC)-I expression levels within the slices, ODC-OVA mice were treated with lipopolysaccharide (LPS; 0.2 mg/kg intraperitoneally) 24 h before slice preparation in a subset of experiments. After 6 h, slices were harvested and embedded in Tissue-Tek OCT compound.

Immunohistochemical staining was performed as previously described [13]. Primary antibodies against neuronal nuclear antigen (NeuN; 1:1,000, Millipore, Schwalbach, Germany), NogoA (1:750; Millipore) and cleaved caspase-3 (1:200, Cell Signaling, Invitrogen) were used. Secondary antibodies were Alexa Fluor 488-coupled goat anti-mouse (1:100, BD Bioscience) and Cy3-coupled goat anti-rabbit (1:300, Dianova, Hamburg, Germany). Negative controls were obtained by either omitting the primary or secondary antibody and revealed no detectable signal (data not shown). For quantification of cell densities, sections were examined using an Axiophot2 microscope (Zeiss, Oberkochen, Germany) equipped with a CCD camera (Visitron Systems, Tuchheim, Germany). Cell density was determined within preselected fields within the cortex.

### Flow cytometry

For flow cytometry analysis of intracellular effector molecule content and activation status of OT-I T cells following incubation in the slice, T cells were retrieved from slices at the end of the incubation period and pooled for each experimental condition. Cells were isolated from the interface of 30% to 50% Percoll (Amersham, Freiburg, Germany) centrifuged for 30 minutes at 2.500 rpm. Mononuclear cells were washed and stained immediately using rat anti-mouse CD8a-PE (BD Bioscience; no. 553033) and rat anti-mouse CD25-FITC (BD Bioscience; no. 554071), rat anti-mouse FoxP3 (ebioscience; 17-5773-80). For intracellular granzyme B staining, cells were pretreated with brefeldin A (BD Bioscience) for 6 h and stained with CD8a-PE followed by an intracellular staining using rabbit anti-mouse granzyme B (ab4059; Abcam, Cambridge, UK) and the secondary antibody goat anti-rabbit Cy2 (Dianova).

### Statistical analysis

All results are presented as mean ± SEM. Statistical analysis was performed using one-way ANOVA with Bonferroni post hoc tests and Student's *t *test modified for small samples [17], as applicable. *P *values < 0.05 were considered significant (indicated as ** in figures).

## Results

### FoxP3^+ ^T reg cells suppress antigen-dependent expansion of naïve CD8^+ ^T cells in a cell-to-cell ratio-dependent manner

**Figure 1:**
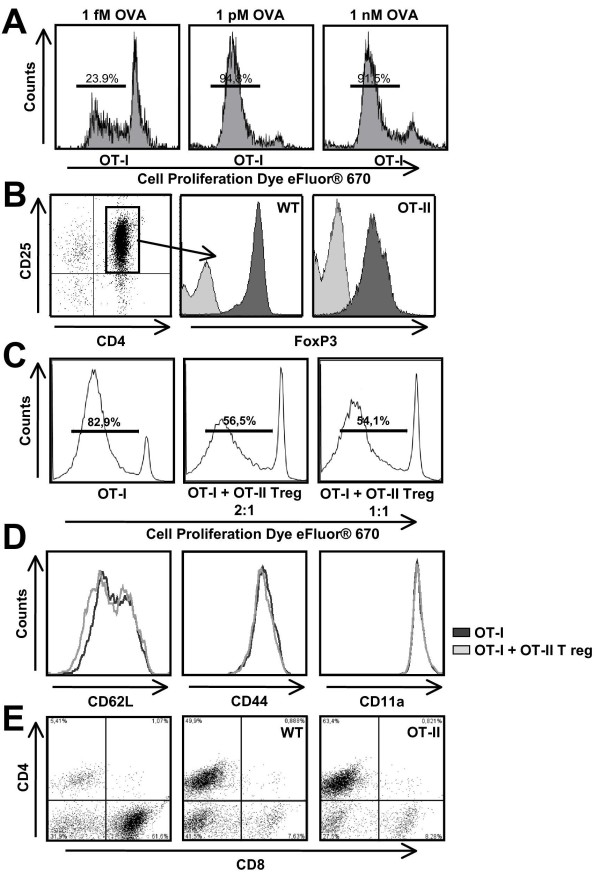
**OT-II regulatory T (T reg) cells suppress antigen-dependent expansion of naïve OT-I T cells. (A) **OVA_257-264 _concentration (1 fM-1 nM) dependence of OT-I splenocyte proliferation assessed after 5 days of stimulation using flow cytometry after prelabeling with Cell Proliferation Dye eFluor 670. **(B) **CD4^+ ^CD25^+ ^T cells from wild-type and OT-II mice show robust forkhead box P3 (FoxP3) expression following polyclonal T cell receptor (TCR) stimulation (CD3) together with costimulation (CD28). Representative flow cytometry analysis of FoxP3 expression levels (dark gray) compared to isotype controls (light gray) in activated CD4^+ ^CD25^+ ^T reg cells (left) from wild-type (middle) and OT-II (right) mice. **(C) **Representative flow cytometry analysis of the reduction of cell proliferation as revealed by prelabeling with Cell Proliferation Dye eFluor 670 upon stimulation of naïve OT-I splenocytes with OVA_257-264 _(0.1 pM) for 5 days in the absence or presence of OT-II T reg cells at different cell-to-cell ratios (2:1 and 1:1). **(D) **Representative flow cytometry analysis of OT-I T cell maturation markers (CD62L, CD44, CD11a) after 5 days of stimulation with OVA_257-264 _(0.1 pM) in the absence or presence of OT-II T reg cells at a cell-to-cell ratio of 2:1. **(E) **Representative flow cytometry analysis of CD4^+ ^and CD8^+ ^cell populations after 5 days of stimulation of OT-I splenocytes with OVA_257-264 _(0.1 pM) in the absence (left) or presence of wild-type (middle) or OT-II (right) T reg cells at a cell-to-cell ratio of 2:1.

Following polyclonal TCR stimulation together with costimulation, T reg cells are known to inhibit expansion of naïve CD4^+ ^and CD8^+ ^T cells upon priming with their cognate antigen [5,6]. To test whether this holds true for our experimental system, OT-I splenocytes were first antigen stimulated for 5 days with the MHC class I-restricted OVA_257-264 _(SIINFEKL) peptide at different concentrations (1 fM to 1 nM), which resulted in a concentration-dependent increase of cell proliferation as revealed by prelabeling with Cell Proliferation Dye eFluor 670 (Figure 1A). Prior to cocultivation with OT-I splenocytes, CD4^+ ^CD25^+ ^T cells were isolated from wild-type and OT-II splenocyte suspensions and stimulated overnight using 1 μg/ml anti-CD3 (immobilized) and 1 μg/ml anti-CD28 (soluble) yielding robust FoxP3 expression levels in both cell populations (Figure 1B). Cocultivation of OT-I splenocytes at an OVA_257-264 _peptide concentration of 0.1 pM with wild-type (data not shown) or OT-II T reg cells (Figure 1C) at different ratios (2:1 and 1:1) resulted in a concentration-dependent reduction of cell proliferation. In contrast, T cell maturation markers (CD62L, CD44, CD11a) were not affected by the presence of wild-type (data not shown) or OT-II T reg cells (Figure 1D). Importantly, non-activated OT II T reg cells and non-activated wild-type T reg cells did not exert any suppressive effect (data not shown). As a control we performed flow cytometry for CD4 and CD8 at the end of the suppression assay. Upon cocultivation with wild-type or OT-II T reg cells viable CD4^+ ^T cells could be detected together with a strong reduction of CD8^+ ^T cells, whereas cultivation of OT-I T cells alone resulted in robust numbers of CD8^+ ^T cells (Figure 1E). This excludes substantial MHC class I-restricted OVA_257-264 _peptide presentation by CD4^+ ^T reg cells and their subsequent killing by activated CD8^+ ^T effector cells.

Hence, following polyclonal TCR stimulation together with costimulation, both wild-type and OT-II T reg cells exert suppressive effects on the antigen-dependent expansion but not maturation of naïve OT-I T cells. This suppression is independent from their own antigen (re)stimulation as neither wild-type nor OT-II T reg cells received additional MHC class II-dependent antigen stimulation during these experiments.

### The strength of the antigen stimulation of CD8^+ ^T effector cells limits the suppressive effect of FoxP3^+ ^T reg cells on cytotoxicity

**Figure 2:**
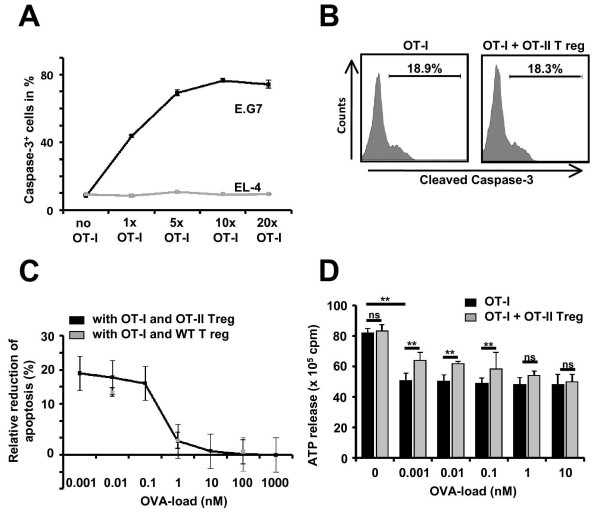
**Suppression of cytotoxicity by OT-II T reg cells depends on the strength of the antigen stimulation of OT-I T cells in a cell coculture system**. (A) Ratio of apoptotic cleaved caspase-3^+ ^EG.7 and EL-4 cells after 6 h of cocultivation with activated OT-I T cells at different ratios in the absence of externally applied OVA_257-264 _(n = 6, respectively). (B) Representative flow cytometry analysis of cleaved caspase-3 levels in EG.7 cells cocultured for 6 h at a ratio of 1:1 with activated OT-I T cells in the absence (left panel) and the presence (right panel) of OT-II T reg cells at a ratio of 2:1 (cleaved caspase-3^+ ^EG.7 cells: no OT-I T cells: 6.5 ± 1.3%; OT-I T cells: 18.9 ± 2.7%; OT-I T cells + OT-II T reg cells: 18.3 ± 3.4%; *P *= 0.864; n = 4). (C) OVA_257-264 _concentration dependence of the OT-II T reg and wild-type T reg cell-mediated reduction of cleaved caspase-3^+ ^EL-4 cells induced by activated OT-I T cells (ratio OT-I to T reg: 2:1; ratio OT-I to EL-4: 1:1). At low OVA_257-264 _concentrations (0.001 to 1 nM) OT-II T reg cells and wild-type T reg cells caused significant reduction of activated OT-I T cell-induced cleaved caspase-3^+ ^EL-4 cells (n = 6, respectively). At high OVA_257-264 _concentrations (10 to 1,000 nM) neither OT-II T reg cells nor wild-type T reg cells exerted any suppressive effect (n = 6, respectively). (D) OVA_257-264 _peptide concentration dependence of intracellular ATP levels (detected following cell lysis) as a measure of cell viability in EL-4 cells following 4 h of cocultivation with activated OT-I T cells (ratio 1:1) in the absence and presence of OT-II T reg cells (ratio 2:1). At low OVA_257-264 _concentrations (0.001 to 0.1 nM) significantly higher intracellular ATP levels in EL-4 cells (ratio 1:1) were detected in the presence of OT-II T reg cells as compared to their absence (OVA_257-264 _0.1 nM: *P *= 0.0271; n = 8). At high OVA concentrations (1 to 10 nM) OT-II T reg cells did not exert any suppressive effect (OVA_257-264 _10 nM: *P *= 0.734; n = 8). *P *values < 0.05 were considered significant (indicated as **).

Activated OT-I T cells induced apoptosis as revealed by positive immunoreactivity for cleaved caspase-3 in a cell-to-cell ratio-dependent manner when cocultured for 6 h with EG.7 target cells constitutively presenting OVA_257-264 _[15] (Figure 2A). In contrast, EL-4 cells, which do not present OVA_257-264 _[16], did not exhibit caspase-3 activation upon exposure to activated OT-I T cells at any cell-to-cell ratio (Figure 2A).

To assess a possible suppressive effect of wild-type and OT-II T reg cells on the cytotoxic activity of activated OT-I T cells, EG.7 cells were cocultured for 6 h at a ratio of 1:1 with activated OT-I T cells alone or together with wild-type or OT-II T reg cells at a ratio of 2:1. Under these conditions wild-type (data not shown) and OT-II T reg cells (Figure 2B) did not significantly alter the number of apoptotic EG.7 cells. Constitutive expression and H-2 K^b^-bound presentation of OVA_257-264 _by EG.7 cells (approximately 90 K^b^:OVA_257-264 _complexes per cell [18,19]) leads to a strong antigen-dependent perforin-granzyme-mediated killing by activated OT-I T cells, which might be hardly overcome by T reg cells under these conditions.

Therefore, we assessed a possible suppressive effect of OT-II T reg cells as well as wild-type T reg cells on OT-I T cell cytotoxicity on EL-4 cells at different OVA_257-264 _concentrations but otherwise unchanged conditions (Figure 2C, D). At low OVA_257-264 _concentrations (0.001 to 1 nM), which lead to no more than 110 K^b^:OVA_257-264 _complexes per cell [20], activated OT-I T cells caused significantly lower numbers of cleaved caspase-3 positive EL-4 cells (ratio 1:1) in the presence of wild-type or OT-II T reg cells (ratio 2:1) as compared to their absence. In contrast, at high OVA_257-264 _concentrations (10 to 1,000 nM), which lead to up to 1,120 K^b^:OVA_257-264 _complexes per EL-4 cell [20], wild-type and OT-II T reg cells (Figure 2C) did not exert any suppressive effect. This was also true when the amount of ATP released upon cell lysis was assessed as a measure of EL-4 cell viability at the end of the incubation period (Figure 2D). Importantly, non-activated OT II T reg cells and non-activated wild-type T reg cells did not exert any suppressive effect (data not shown). This excludes any unspecific effect resulting from increased total cell numbers after adding T reg cells in these experiments. Moreover, in the total absence of OVA_257-264_, there was a significantly higher EL-4 cell viability at the end of the incubation period, that is, no killing and therefore no suppressive effect of T reg cells (Figure 2D).

These results are consistent with the known high antigen affinity of OT-I T cells, where as few as 1 to 3 K^b^:OVA_257-264 _complexes per target cell are sufficient to induce cytotoxicity [14,21,22]. This illustrates the fact that the suppressive effect of natural CD4^+ ^T reg cells depends on the strength of this antigen stimulus delivered to the CD8^+ ^T effector cell population. Moreover, T reg cells exert their suppressive effect independent from their own antigen (re)stimulation as neither wild-type nor OT-II T reg cells received additional MHC class II-dependent antigen stimulation during these experiments.

### FoxP3^+ ^T reg cells reduce CD8^+ ^T cell-induced antigen-specific neural damage in acute brain slices

To assess whether T reg cells exert their suppressive effect on OT-I T cell-mediated cytotoxicity also in a more physiological environment, we incubated activated OT-I T cells in the absence and presence of OT-II T reg cells or wild-type T reg cells at a ratio of 2:1 for 6 h in acute brain slices from ODC-OVA mice. These mice selectively express ovalbumin under the control of a truncated MBP promoter as a neo-self antigen in ODCs [11]. Incubation of activated OT-I T cells results in an ODC-directed CD8^+ ^T cell attack in ODC-OVA slices, which leads to perforin-granzyme-dependent death of ODCs and collateral death of neurons [13]. As these ODCs exhibit approximately 20 K^b^: OVA_257-264 _complexes per cell [20], a suppressive effect of T reg cells on OT-I T cell cytotoxicity seems conceivable according to our cell culture experiments (given comparable ratios of OT-I T cells, T reg cells and ODC target cells within the slice).

**Figure 3:**
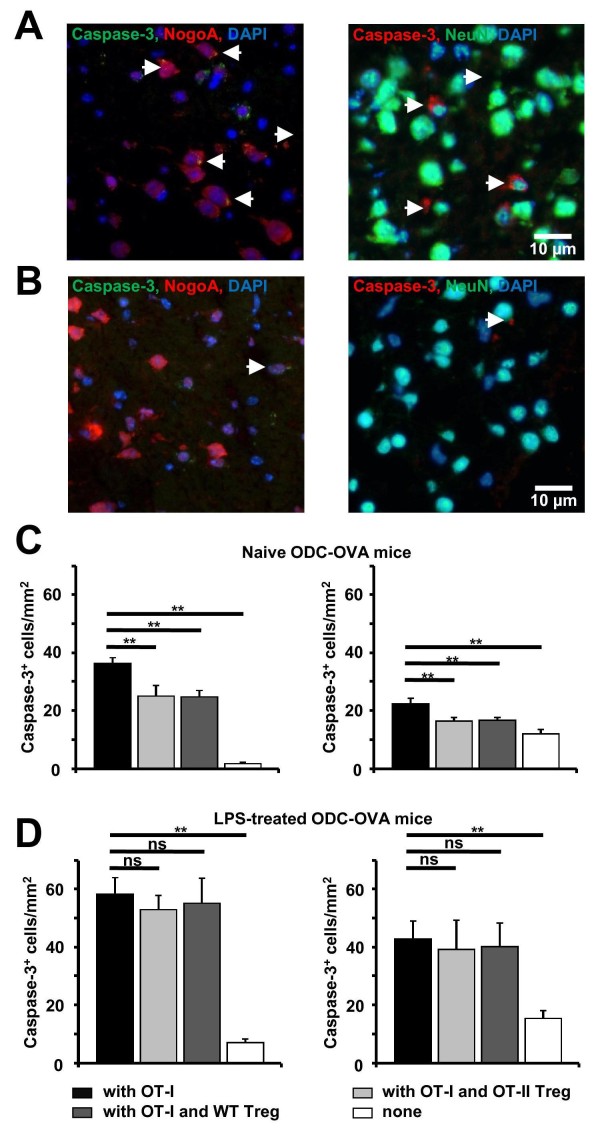
**OT-II regulatory T (T reg) cells reduce OT-I T cell-induced oligodendroglial (and collateral neuronal) apoptosis in acute brain slices form ODC-OVA mice**. (A, B) Representative immunofluorescence images of the cortical gray matter of ODC-OVA slices incubated for 6 h with (A) or without (B) activated OT-I T cells. Apoptotic NogoA^+ ^ODCs (left) and neuronal nuclear antigen (NeuN)^+ ^neurons (right) were detected by staining for cleaved caspase-3. (C) Densities of cleaved caspase-3^+ ^NogoA^+ ^ODCs (left) and NeuN^+ ^neurons (right) in slices from naïve ODC-OVA mice under the conditions indicated (n = 6, respectively). *P *values < 0.05 were considered significant (indicated as **). (D) Densities of cleaved caspase-3^+ ^NogoA^+ ^ODCs (left) and NeuN^+ ^neurons (right) in slices from ODC-OVA mice treated intraperitoneally with lipopolysaccharide (LPS) (0.2 mg/kg) 24 h before preparation of brain slices under the conditions indicated (n = 5, respectively). *P *values < 0.05 were considered significant (indicated as **).

In slices from naïve ODC-OVA mice, incubation of activated OT-I T cells together with wild-type or OT-II T reg cells at a ratio of 2:1 resulted in a significantly reduced density of cleaved caspase-3^+ ^NogoA^+ ^ODCs as compared to incubation of OT-I T cells alone (Figure 3A-C, left panels).

During the ODC-directed OT-I T cell attack, neurons in the gray matter of ODC-OVA slices undergo collateral apoptosis [13]. In the presence of OT-II T reg cells or wild-type T reg cells, OT-I T cell-mediated collateral neuronal damage as revealed by the density of cleaved caspase-3^+ ^NeuN^+ ^neurons was also significantly reduced (Figure 3A-C, right panels). Notably, background levels of cleaved caspase-3^+ ^neurons and ODC are significantly lower than those evoked by OT-I T cell incubation (Figure 3B) and background levels are known to be higher in neurons than in ODCs [13] (Figure 3C).

Intraperitoneal treatment of mice with LPS is known to augment MHC I expression levels and thus antigen-peptide presentation on neural cell within 24 h [23]. Hence, to assess how T reg cell-mediated suppression of OT-I T cell cytotoxicity in ODC-OVA slices relates to MHC I expression, ODC-OVA mice were intraperitonally treated with LPS (0.2 mg/kg) 24 h before brain slice preparation. Consistent with a LPS-enhanced MHC I expression, densities of cleaved caspase-3^+ ^ODCs and neurons were significantly enhanced upon exposure to activated OT-I T cells (with similar background levels) in ODC-OVA slices from LPS-treated as compared to untreated mice (Figure 3D, left and right panels). Consistent with previous data on the dependence of T reg cell-mediated suppression on the antigen stimulus delivered to the CD8^+ ^T effector cell, we observed no significant reduction of cell death of ODCs and collateral death of neurons by T reg cells under these conditions (Figure 3D, left and right panels).

**Figure 4:**
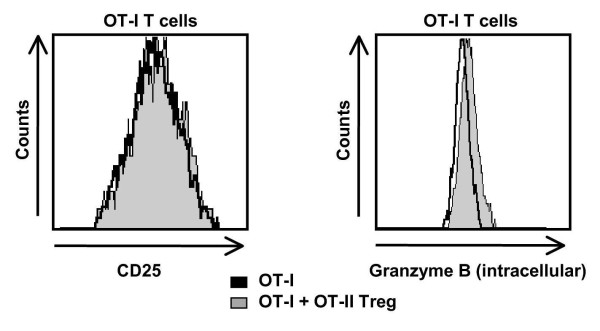
**OT-II regulatory T (T reg) cells reduce release of cytotoxic effector molecules from OT-I T cells in acute brain slices form ODC-OVA mice**. Representative flow cytometry analysis of activation markers (CD25, left panel) and intracellular effector molecules (granzyme B, right panel) of OT-I T cells retrieved and pooled from ODC-OVA slices incubated with activated OT-I T cells alone or together with OT-II T reg cells. Mean fluorescence intensities (MFI) of pooled OT-I T cells from slices incubated in the absence and presence of OT-II T reg cells were 455 vs 478 for CD25 (left panel) and 63 vs 79 for intracellular granzyme B (right panel).

At the end of the incubation period, T cells were harvested from the slice, pooled and analyzed by flow cytometry. Irrespective of the presence of OT-II T reg cells or wild-type T reg cells (data not shown), harvested OT-I T cells displayed similar levels of CD25 expression as marker of their activation (Figure 4, left panel). However, staining for the intracellular content of CD8^+ ^T cell effector molecules (that is, granzyme B) revealed a reduced release of effector molecules from OT-I T cells consistent with the reduced levels of apoptotic ODCs and neurons in the presence as compared to the absence of T reg cells (Figure 4, right panel).

## Discussion

To what extent T reg cells traffic to and exert their suppressive effects in parenchymal organs under autoimmune conditions is currently under debate [1]. It was recently reported that, following their expansion in the peripheral lymphoid compartment [24], T reg cells accumulate and further expand in the inflamed CNS of mice undergoing experimental autoimmune encephalomyelitis (EAE). However, T reg cells isolated from the CNS of EAE mice dampened expansion of naïve but not activated encephalitogenic CD4^+ ^T cells [25]. Other studies found accumulation, activation and proliferation of T reg cells within the CNS during murine EAE, which was required for the resolution of inflammation and amelioration of the clinical disease [26,27]. Moreover, application of myelin-reactive T reg cells could suppress the initiation and reverse established myelin antigen-induced EAE in mice [28]. In these studies, up to 50% of CNS invasive CD4^+ ^T cells could be T reg cells [27]. However, in all these experimental systems pathology is mainly driven by CD4^+ ^T cells.

Recently, CD8^+ ^T cells have emerged as key players in autoimmune neuroinflammation, considered to have specific relevance for exertion of parenchymal damage [12]. Hence, we directly tested the capability of CD4^+ ^T reg cells to suppress terminal effector function of activated CD8^+ ^T cells both in cell culture as well as within intact CNS tissue [12]. Given a homogenous CD8^+ ^T cell receptor repertoire and a fixed ratio of effector-to-regulatory T cells, CD4^+ ^T reg cells are capable of limiting cytotoxicity of CD8^+ ^T cells. The suppressive effect of CD4^+ ^T reg cells at the effector site depends on the strength of the antigen signal of the target cells delivered to the CD8^+ ^T effector cells as determined mainly by the number of antigen peptide-loaded MHC I molecules exposed on the target cell surface [20]. The higher the OVA_257-264 _antigen load of EL-4 cell (which express MHC I molecules in excess; [16,20]) or the higher the expression of (OVA_257-264 _antigen loaded) MHC I molecules by ODCs [20] in ODC-OVA slices, the weaker the relative suppressive effect of OT-II or wild-type T reg cells on the action of OT-I T effector cells. Notably, to acquire their suppressive capacity CD4^+ ^T reg cells required activation via their T cell receptor together with a costimulatory signal, but following activation, suppression did not require restimulation and was antigen non-specific in good agreement with earlier results [29,30].

In line with previous data obtained from lymph nodes, CD4^+ ^T reg cells did not alter activation status but functionally impaired the release of cytotoxic granules resulting in higher intracellular levels of effector molecules in CD8^+ ^T cells at the effector site [31]. As a note of caution one should consider that our experimental systems do not allow an exact estimation to what extent parenchymal T reg cell-mediated T effector cell suppression influences the course and severity of systemic experimental CNS inflammation. When considering the therapeutic potential in CNS inflammation, this component would certainly be of high relevance (see for example, [1]).

## Conclusions

Taken together our report demonstrates that CD4^+ ^T reg cells are able to limit cytotoxicity of an ongoing CD8^+ ^T effector cell attack within the intact CNS parenchyma. This effect depends on the strength of the antigen signal delivered to the CD8^+ ^T effector cells and (presumably) the ratio of regulatory to effector T cells.

## Competing interests

The authors declare that they have no competing interests.

## Authors' contributions

KG, SB, NM, AD, SP and MKS acquired, analyzed and interpreted data; NM, SGM and HW wrote and revised the manuscript. All authors read and approved the final manuscript.
